# Tannic Acid Extracted from *Galla chinensis* Supplementation in the Diet Improves Intestinal Development through Suppressing Inflammatory Responses via Blockage of NF-κB in Broiler Chickens

**DOI:** 10.3390/ani12182397

**Published:** 2022-09-13

**Authors:** Changwei Jing, Jiaxing Niu, Yang Liu, Ning Jiao, Libo Huang, Shuzhen Jiang, Lei Yan, Weiren Yang, Yang Li

**Affiliations:** 1 Shandong Provincial Key Laboratory of Animal Biotechnology and Disease Control and Prevention, Department of Animal Science and Veterinary Medicine, Shandong Agricultural University, Daizong Street 61#, Tai’an 271018, China; 2 College of Animal Science and Veterinary Medicine, Huazhong Agricultural University, Shizishan Street 1#, Wuhan 430070, China; 3 Shandong New Hope Liuhe Group Co., Ltd., Jiudongshui Road 592-26#, Qingdao 266100, China

**Keywords:** chicken, feed additives, intestine, plant extract, tannic acid

## Abstract

**Simple Summary:**

Tannic acid (TA) is a hydrophilic polyphenolic compound present in most plants. *Galla chinensis*, a traditional Chinese medicine, is widely available and used in China, and TA is one of the main bioactive components of *Galla chinensis*. Until now, there is little information available in the scientific literature on the evaluation of the application of *Galla chinensis* TA in broiler production. A healthy and well-developed intestine is critical in absorbing nutrients and serves as a vital barrier against pathogen invasion. However, the intestine of the broiler is vulnerable to inflammatory damage under intensive poultry farming conditions. Therefore, we aimed to investigate the effects of adding *Galla chinensis* TA to the diet of broiler chickens on intestinal development and health, and highlight the application of TA sourced from *Galla chinensis* as a potential feed additive in poultry production. Our results showed that 300 mg/kg microencapsulated TA from *Galla chinensis* supplementation benefited to improve intestinal development, which might be attributed to the suppression of inflammatory responses via blockage of NF-κB in broiler chickens, and provide guidance for the application of *Galla chinensis* TA in poultry production.

**Abstract:**

The objective of this study was to investigate the effects of adding tannic acid (TA) extracted from *Galla chinensis* to the diet of broiler chickens on intestinal development. A total of 324 healthy 1-day-old broilers were used in a 42 d study, and divided into two treatment groups at random (six replicates per group). Broilers were either received a basal diet or a basal diet supplemented with 300 mg/kg microencapsulated TA extracted from *Galla chinensis*. The results showed that dietary supplemented with 300 mg/kg TA from *Galla chinensis* improved intestinal morphology, promoted intestinal mucosal barrier integrity, and elevated mucosal expressions of nutrients transporters and tight junction protein *CLDN3* in broilers. Besides, 300 mg/kg TA from *Galla chinensis* supplementation decreased the concentrations of inflammatory cytokines in serum and intestinal mucosa and reduced the mRNA expression of NF-κB in intestinal mucosa. Above all, supplementation of 300 mg/kg microencapsulated TA extracted from *Galla chinensis* showed beneficial effects in improving intestinal development, which might be attributed to the suppression of inflammatory responses via blockage of NF-κB in broiler chickens. These findings will support the use of TA sourced from *Galla chinensis* in poultry industry.

## 1. Introduction

A well-developed intestine serves as a crucial barrier against pathogen invasion as well as being essential in digesting and absorbing nutrients [1]. However, due to pathogenic bacteria, environmental variables, and poor feed hygiene, broiler chickens are at an increased risk of developing intestinal disorders as a result of the rapid global spread of intensive farming [2,3]. Previous research has shown that intestinal disorders can limit intestinal development, disrupt intestinal digestion and absorption, and induce an imbalance in the intestinal flora, which can result in growth restriction, disease, and even death in broiler chickens [4,5]. With the prohibition of antibiotic growth promoters due to its significant negative impacts on environmental conditions and human health, it is critical need to find new feed additives that promote intestinal health and development in poultry production [6].

Plant extracts, such as tannic acids, have shown success in positively influencing the intestinal development of poultry [7,8]. Tannic acid from plants is typically divided into hydrolysable tannic acid (TA) and condensed TA due to its variable chemical structure, and reported to have numerous biological functions, such as anti-inflammatory, antioxidant, anti-bacterial, and apoptosis regulation [9]. *Galla chinensis* is a traditional Chinese medicine, and is widely available and used in China. One of the main bioactive components of *Galla chinensis* is hydrolysable TA, which is a glycoside or ester formed by the combination of gallic acid and diacid with glucose called gallotannins [10]. Tannic acid was once thought of an anti-nutritional feed additive for monogastric animals due to their binding properties with carbohydrates, proteins, minerals, and digestive enzymes, leading to the decrease of bioavailability of nutrients in feed [11,12]. Recent studies showed that supplementing low concentrations of TA to the diet could improve growth performance and promote the liver and intestinal health of animals [8], suggesting that TA could be a good feed additive for animal production [13,14]. However, the majority of TA is generally derived from chestnut and quebracho, which is a polymeric molecule called ellagitannins that is formed by ellagic acid conjugated with a glycoside moiety [15], and few studies have investigated the *Galla chinensis* TA as the feed additive in poultry production. Wang et al. [16] showed that 500 to 1000 mg/kg TA from *Galla chinensis* addition had beneficial effects on intestinal morphology, nutrient transport, and microbiota composition in weaning piglets. Song et al. [14] also indicated that 0.1% and 0.2% TA extracted form *Galla chinensis* prevented post-weaning diarrhea and improved intestinal health of weaned piglets. However, there are few detailed studies about whether TA from *Galla chinensis* can promote intestinal development of broiler chickens.

Therefore, this study aimed to investigate the effects of supplementing TA extracted from *Galla chinensis* to the diet of broiler chickens on intestinal development, and provide a reference for the use of *Galla chinensis* TA in poultry industry.

## 2. Materials and Methods

### 2.1. Animals and Diets

A total of 324 healthy 1-day-old Arbor Acres broilers with similar initial weight (48.35 ± 0.41 g) were used in a 42-d study. All broilers were divided into two treatment groups at random (six replicates per group and 27 broiler chickens each replicate) and housed in three-level wired cages. The cages were placed in a light- and temperature-controlled room with consistent illumination. The broiler chickens either received a basal diet (control (CON) group) or a basal diet supplemented with 300 mg/kg TA extracted from *Galla chinensis* (TA group). The basal diets (Table 1) were formulated based on nutrient requirements of the National Research Council (NRC, 1994). The TA production (40% effective concentrations) was microencapsulated to improve the palatability [8,16], and provided by the Wufeng Chicheng Biotechnology Co., Ltd. (Yichang, China) by whom the dosage for *Galla chinensis* TA supplementation in broiler diet was recommended. The technology of microencapsulation was provided by the Kangjude Company Ltd. (Hangzhou, China) as previously described in Wang et al. [16]. The broilers were fed according to a two-phase feeding program (0–21 d and 21–42 d), and had free access to feed and water throughout the experiment. A Newcastle disease vaccine and an inactivated infectious bursal disease vaccine were inoculated on days 7 and 14 of the trial, respectively. The temperature of the room was maintained at 35 °C at the first week, and then gradually reduced to 21 °C at the rate of 0.5 °C daily.

### 2.2. Sampling Procedure

On day 42 of trial, a total of twelve broilers (one broiler from each replicate with similar weight to the cage average) were selected to collect intestinal tissue samples after being narcotized by CO_2_ asphyxiation. About 2 cm length segments cut from the medium of the small intestine were flushed gently with a 0.9% saline solution and fixed in 4% paraformaldehyde for 24 h. Then, mucosal tissue was carefully scraped with a sterile glass slide from the medium of the small intestine after being washed with an ice-cold saline solution, and chilled in liquid nitrogen. Finally, the mucosal tissue samples were stored at −80 °C for further analysis.

### 2.3. Determination of Serum Inflammatory Cytokines, D-Lactate, and Diamine Oxidase (DAO) Concentrations

Serum inflammatory cytokines including interleukin-1beta (IL-1β), tumor necrosis factor-alpha (TNF-α), interleukin-10 (IL-10), and interleukin-6 (IL-6), as well as D-lactate and DAO, were determined with the specific ELISA kits (Jiangsu Meimian Industrial Co., Ltd., Jiangsu, China) as previously described [17].

### 2.4. Measurements of Intestinal Morphology

The fixed intestinal segments were dehydrated in ethanol and xylene solutions and embedded according to conventional paraffin-embedding protocol. Then, the slices were processed by hematoxylin and eosin staining after being cut into 5 μm thin slices using Leica semi-automatic microtome (Leica Co., Wetzlar, Germany). The intestinal morphology was examined, and villus height (VH), crypt depth (CD), and VH/CD ratio were analyzed according to the method described in Chen et al. [18].

### 2.5. Determination of Intestinal Mucosal Barrier Factors and Total Protein Concentrations

Intestinal mucosal barrier factors including zonula occludens-1 (ZO-1), trefoil factor family (TFF), mucin 2 (MUC2), and transforming growth factor-alpha (TGF-α) were determined with the specific ELISA kits (Jiangsu Meimian) [17]. The concentrations of ZO-1, TFF, MUC2, and TGF-α were normalized to each sample’s total protein concentration, which was quantified with a BCA protein assay (Jiangsu Meimian).

### 2.6. Determination of Intestinal Mucosal Inflammatory Cytokines and SIgA Concentrations

The intestinal mucosal concentrations of IL-1β, IL-6, IL-10, TNF-α, and secretory immunoglobulin A (SIgA) were examined with ELISA kits (R&D Systems Inc., Minneapolis, MN, USA) according to the detection steps of ELISA operation described in Chen et al. [18]. Inflammatory cytokines and SIgA concentrations in intestinal mucosa were normalized to each sample’s total protein concentration.

### 2.7. Determination of Relative mRNA Expression in Intestinal Mucosa

The mucosa mRNA expression levels of tight junction proteins [(ZO-1, occludin (OCLN), claudin 2 (CLDN2), and claudin 3 (CLDN3)], nutrient transporters [(glucose transporter type 2 (GLUT2), Na+/glucose cotransporter (SGLT1), y+L amino acid transporter 1 (y+LAT1), cationic amino acid transporter 1 (CAT1), and fatty acid binding protein-1 (FABP1)], and inflammatory pathway genes [(Toll-like receptor 4 (TLR4), myeloid differentiation primary response 88 (MyD88), and nuclear factor-kappa B (NF-κB)] in intestinal samples were determined using a CFX-96 real-time PCR detection system (Bio-Rad, Hercules, CA, USA). The primer sequences used for real-time PCR and detailed procedure of the relative mRNA expression determination in this study refer to previous studies [17,19]. The β-actin gene was amplified in parallel as the internal control for gene normalization and quantification. The 2^−ΔΔ^ Ct method was used to calculate the relative mRNA abundances of target genes in intestinal mucosa samples.

### 2.8. Statistical Analyses

The individual chicken data were used to access the effects on all variables. Statistical analyses for all data were performed with *t*-test of SAS (9.4 Inst. Inc., Cary, NC, USA) after using the Shapiro–Wilk W statistic to check normality of the data. Values are expressed as mean ± standard error. Statistical significance was set at *p* < 0.05, and a trend toward significance was considered at 0.05 ≤ *p* < 0.10.

## 3. Results

### 3.1. Serum Inflammatory Cytokines, D-Lactate, and DAO

**Figure 1:**
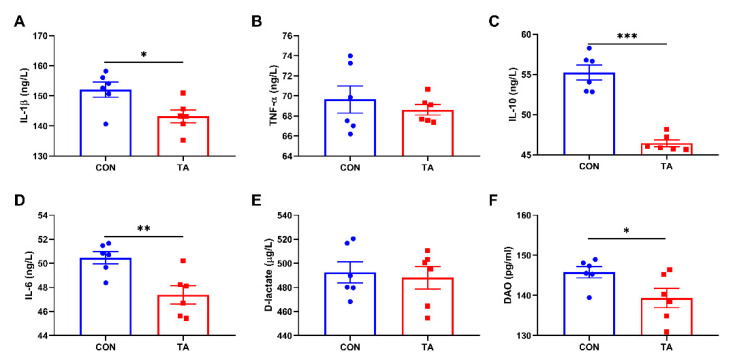
Effects of tannic acid extracted from *Galla chinensis* supplementation on serum concentrations of inflammatory cytokines, D-Lactate, and diamine oxidase in broiler chickens. (**A**) Interleukin-1 beta (IL-1β); (**B**) Tumor necrosis factor-alpha (TNF-α); (**C**) Interleukin-10 (IL-10); (**D**) Interleukin-6 (IL-6); (**E**) D-lactate; (**F**) Diamine oxidase (DAO). CON, broiler chickens fed basal diet; TA, broiler chickens fed basal diet supplemented with 300 mg/kg microencapsulated tannic acid extracted from *Galla chinensis*. Values are mean ± standard error (*n* = 6). Differences between treatments were displayed by * *p* < 0.05, ** *p* < 0.01, and *** *p* < 0.001.

As shown in Figure 1, dietary supplementation with 300 mg/kg TA from *Galla chinensis* significantly decreased (*p* < 0.05) IL-1β (Figure 1A), IL-10 (Figure 1C), IL-6 (Figure 1D), and DAO levels (Figure 1F) in intestinal mucosa. There were no significant differences (*p* > 0.05) in serum TNF-α (Figure 1B) and D-lactate (Figure 1E) between broilers fed the CON diet and the TA diet.

### 3.2. Intestinal Morphology

**Figure 2:**
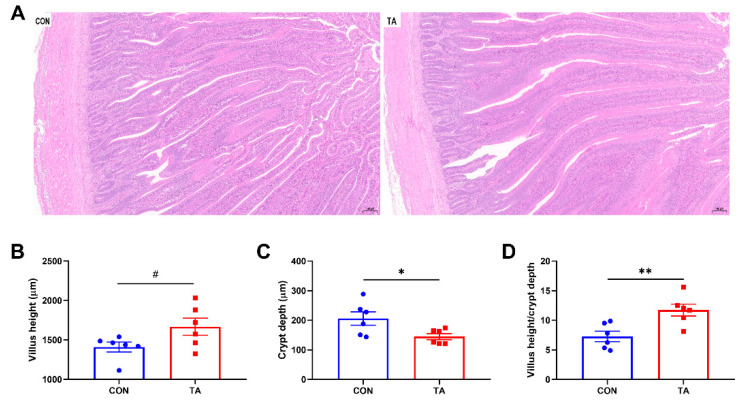
Effects of tannic acid extracted from *Galla chinensis* supplementation on intestinal morphology in broiler chickens. (**A**) Hematoxylin and eosin photomicrographs obtained at 100× magnification; (**B**) Villus height; (**C**) Crypt depth; (**D**) Villus height/crypt depth ratio. CON, broiler chickens fed basal diet; TA, broiler chickens fed basal diet supplemented with 300 mg/kg microencapsulated tannic acid extracted from *Galla chinensis*. Values are mean ± standard error (*n* = 6). Differences between treatments were displayed by # 0.05 ≤ *p* < 0.10, * *p* < 0.05, and ** *p* < 0.01.

The effects of TA supplementation on intestinal morphology are displayed in Figure 2. Compared with CON group, TA group showed thicker and denser villus (Figure 2A). Broilers fed the diet supplemented with TA had significantly decreased (*p* < 0.05) intestinal CD (Figure 2C) and increased (*p* < 0.05) VH/CD ratio (Figure 2D), and tended to have increased (*p* < 0.10) VH (Figure 2B) compared with broilers fed the CON diet.

### 3.3. Intestinal Mucosal Barrier Maturity and Integrity

**Figure 3:**
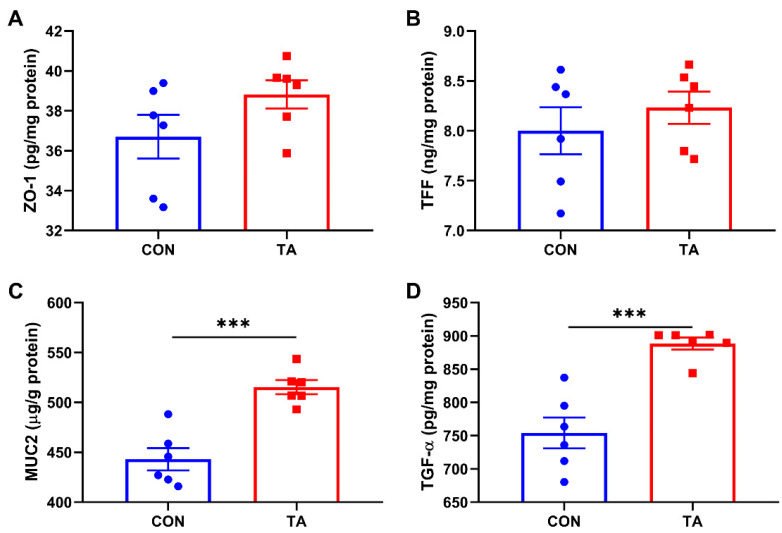
Effects of tannic acid extracted from *Galla chinensis* supplementation on the maturity and integrity of intestinal mucosal barrier in broiler chickens. (**A**) Zonula occludens-1 (ZO-1); (**B**) Trefoil factor family (TFF); (**C**) Mucin 2 (MUC2); (**D**) Transforming growth factor-α (TGF-α). CON, broiler chickens fed basal diet; TA, broiler chickens fed basal diet supplemented with 300 mg/kg microencapsulated tannic acid extracted from *Galla chinensis*. Values are mean ± standard error (*n* = 6). Differences between treatments were displayed by *** *p* < 0.001.

As shown in Figure 3, dietary supplementation with 300 mg/kg TA from *Galla chinensis* significantly increased (*p* < 0.05) MUC2 (Figure 3C) and TGF-α (Figure 3D) concentrations in intestinal mucosa. No significant differences (*p* > 0.05) were observed in intestinal mucosal ZO-1 (Figure 3A) and TFF (Figure 3B) concentrations between the two groups.

### 3.4. Intestinal Mucosal Inflammatory Cytokines and SIgA Concentrations

**Figure 4:**
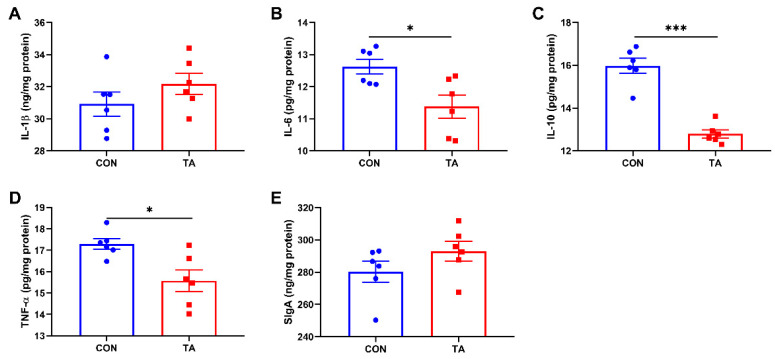
Effects of tannic acid extracted from *Galla chinensis* supplementation on intestinal mucosal inflammatory cytokines and SIgA concentrations in broiler chickens. (**A**) Interleukin-1beta (IL-1β); (**B**) Interleukin-6 (IL-6); (**C**) Interleukin-10 (IL-10); (**D**) Tumor necrosis factor-alpha (TNF-α); (**E**) Secretory immunoglobulin A (SIgA). CON, broiler chickens fed basal diet; TA, broiler chickens fed basal diet supplemented with 300 mg/kg microencapsulated tannic acid extracted from *Galla chinensis*. Values are mean ± standard error (*n* = 6). Differences between treatments were displayed by * *p* < 0.05 and *** *p* < 0.001.

Intestinal mucosal inflammatory cytokines and SIgA concentrations are shown in Figure 4. Dietary supplemented with 300 mg/kg TA from *Galla chinensis* significantly decreased (*p* < 0.05) IL-6 (Figure 4B), IL-10 (Figure 4C), and TNF-α (Figure 4D) concentrations in intestinal mucosa. No significant differences (*p* > 0.05) were observed in intestinal mucosal IL-1β (Figure 4A) and SIgA (Figure 4E) concentrations between the two groups.

### 3.5. Genes Expressions in Intestinal Mucosa

#### 3.5.1. Expressions of Tight Junction Proteins in Intestinal Mucosa

**Figure 5:**
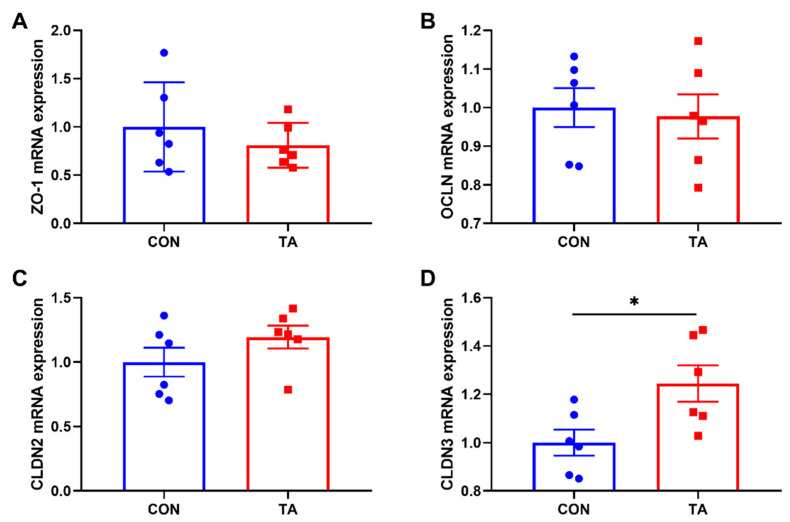
Effects of tannic acid extracted from *Galla chinensis* supplementation on expressions of tight junction proteins in intestinal mucosa of broiler chickens. (**A**) Zonula occludens-1 (ZO-1); (**B**) Occludin (OCLN); (**C**) Claudin 2 (CLDN2); (**D**) Claudin 3 (CLDN3). CON, broiler chickens fed basal diet; TA, broiler chickens fed basal diet supplemented with 300 mg/kg microencapsulated tannic acid extracted from *Galla chinensis*. Values are mean ± standard error (*n* = 6). Differences between treatments were displayed by * *p* < 0.05.

As shown in Figure 5, dietary supplemented with 300 mg/kg TA from *Galla chinensis* significantly increased (*p* < 0.05) CLDN3 (Figure 5D) mRNA expression. No significant differences were observed (*p* > 0.05) in mRNA expressions of ZO-1 (Figure 5A), OCLN (Figure 5B), and CLDN2 (Figure 5C) in small intestine between CON and TA groups.

#### 3.5.2. Expressions of Nutrient Transporters Genes in Intestinal Mucosa

**Figure 6:**
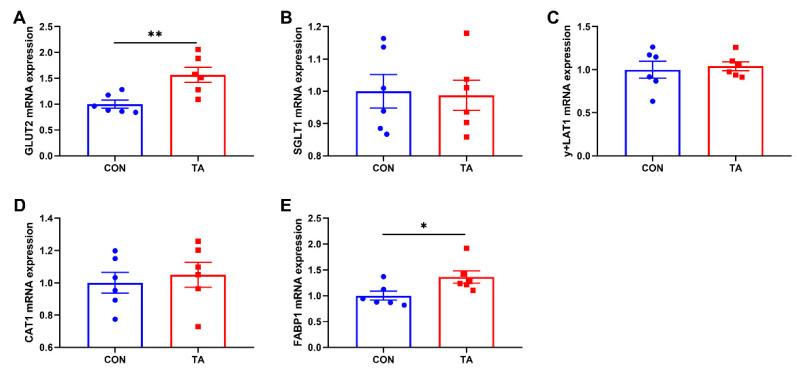
Effects of tannic acid extracted from *Galla chinensis* supplementation on expressions of nutrient transporters genes in intestinal mucosa of broiler chickens. (**A**) Glucose transporter type 2 (GLUT2); (**B**) Na+/glucose cotransporter (SGLT1); (**C**) y+L amino acid transporter 1 (y+LAT1); (**D**) Cationic amino acid transporter 1 (CAT1); (**E**) Fatty acid binding protein-1 (FABP1). CON, broiler chickens fed basal diet; TA, broiler chickens fed basal diet supplemented with 300 mg/kg microencapsulated tannic acid extracted from *Galla chinensis*. Values are mean ± standard error (*n* = 6). Differences between treatments were displayed by * *p* < 0.05 and ** *p* < 0.01.

As shown in Figure 6, dietary TA supplementation significantly increased (*p* < 0.05) GLUT2 (Figure 6A) and FABP1 (Figure 6E) mRNA expressions in intestinal mucosa. There were no significant differences (*p* > 0.05) in the mRNA expressions of SGLT1 (Figure 6B), y+LAT1 (Figure 6C), and CAT1 (Figure 6D) in intestinal mucosa between CON and TA groups.

#### 3.5.3. Expressions of Inflammatory Pathway Genes in Intestinal Mucosa

**Figure 7:**
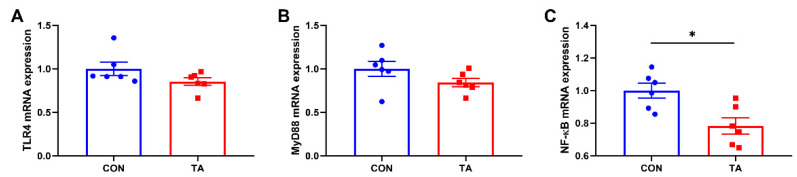
Effects of tannic acid extracted from *Galla chinensis* supplementation on expressions of inflammatory pathway genes in intestinal mucosa of broiler chickens. (**A**) Toll-like receptor 4 (TLR4); (**B**) Myeloid differentiation primary response 88 (MyD88); (**C**) Nuclear factor-kappa B (NF-κB). CON, broiler chickens fed basal diet; TA, broiler chickens fed basal diet supplemented with 300 mg/kg microencapsulated tannic acid extracted from *Galla chinensis*. Values are mean ± standard error (*n* = 6). Differences between treatments were displayed by * *p* < 0.05.

As shown in Figure 7, TA group showed significantly lower (*p* < 0.05) mucosal NF-κB (Figure 7C) mRNA expression than CON group. No significant differences were observed (*p* > 0.05) in TLR4 (Figure 7A) and MyD88 (Figure 7B) expressions between CON and TA groups.

## 4. Discussion

The small intestine is the principal organ in charge of nutrient absorption, and the crypt-villus structure is responsible for efficient nutrition intake [20]. In the present study, 300 mg/kg TA from *Galla chinensis* supplementation in the diet increased VH and VH/CD ratio and decreased CD of small intestine. Previous studies in weaned piglets also demonstrated that TA from *Galla chinensis* supplementation increased intestinal VH and VH/CD ratio and decreased intestinal CD [14,16]. The higher VH and VH/CD are related to enhanced digestion and absorption capacity of the intestine [18]. The results of present study might suggest that dietary 300 mg/kg TA from *Galla chinensis* supplementation benefited to the digestion and absorption of nutrients. Consistently, we also found that TA group had higher intestinal expressions of GLUT2 and FABP1 than CON group in current study. Facilitative glucose transporters play a vital role in glucose utilization. Glucose transporter type 2, encoded by the SLC2A2 gene, is a bidirectional glucose transporter presented in the plasma membrane of the liver, intestine, kidney, and pancreas, and is an essential component of intestinal glucose sensing and incretin secretion [21,22]. Intestinal FABP1 is a small intracellular lipid-binding protein. Previous study in mice showed that deficiency of FABP1 lead to divergent changes in intestinal lipid transport and metabolism and whole-body energy homeostasis [23]. Wang et al. [16] demonstrated that TA from *Galla chinensis* supplementation increased the expressions of nutrient transporters SLC6A19 and SLC15A1 in small intestine of weaned piglets. Therefore, it indicated that 300 mg/kg TA from *Galla chinensis* supplementation helped to improve intestinal morphology and enhance intestinal digestion and the absorption capacity of broilers in this study.

Barrier integrity, as well as intestinal morphology, is another crucial index of intestinal development [20,24]. In current study, 300 mg/kg TA from *Galla chinensis* supplementation reduced serum DAO activity. Diamine oxidase is an intracellular enzyme for the metabolism of ingested histamine, and primarily synthesized by enterocytes [25]. Once the intestinal mucosal is damaged, intestinal permeability will increase, and the intestinal DAO will be released into the blood circulation [26]. Hence, the serum level of DAO is a marker for evaluating the extent of the intestinal mucosa injury. Previous study in weaned piglets also showed that dietary 0.2% TA supplementation reduced serum DAO activity [27]. The decreased serum levels of DAO might demonstrate that dietary TA from *Galla chinensis* supplementation benefited to maintain the integrity of intestinal mucosa. Besides, we also found that TA from *Galla chinensis* supplementation increased concentrations of MUC2, TGF-α and mRNA expression of CLDN3 in small intestinal mucosa in the current study. Mucin 2 is mostly produced by the goblet cells and is the main mucin that located throughout the surface of the intestinal epithelium [28]. Recent studies demonstrated that MUC2 is involved in intestinal barrier protection and microbiome homeostasis modulation [28,29]. Transforming growth factor-alpha is a potent growth factor and belongs to the epidermal growth factor family. It was reported that TGF-α is produced by the intestinal epithelial cell and capable of maintaining the integrity of intestinal epithelial cells [30]. In the intestine, CLDN3 is thought to be the important sealing protein, and plays an important role in maintaining the intestinal barrier and defending against systemic inflammatory diseases [31]. The loss of CLDN3 is an early event in the development of intestinal damage [32]. The study in weaned piglets also indicated that TA from *Galla chinensis* supplementation increased mRNA expression levels of tight junction proteins including ZO-1, ZO-2, and CLDN3 [14]. Above all, dietary 300 mg/kg TA from *Galla chinensis* supplementation could promote intestinal mucosal barrier integrity in broiler chickens in the present study.

Inflammatory response is usually associated with intestinal maldevelopment and mucosal barrier damage [33]. In the present study, dietary TA supplementation decreased serum concentrations of IL-1β, IL-6, and IL-10, and reduced small intestinal mucosal IL-6, IL-10, and TNF-α concentrations in broilers. Interleukin-6, IL-10, IL-1β, and TNF-α are four major pro-inflammatory cytokines implicated in the pathogenesis of many inflammatory-associated diseases. Interleukin-1beta is a key mediator of inflammation, contributing to the development of many diseases. It also plays a critical part in defending cells against endogenous stress and microbial pathogen infection [34]. During acute or chronic inflammation, TNF-α is generated by macrophages/monocytes, and responsible for a wide range of signaling events within cells that result in necrosis or apoptosis [35]. Moreover, IL-1β and TNF-α both can activate the generation of pro-inflammatory IL-6 and then contributes to the pathogenesis of various diseases, such as inflammation, autoimmunity, and malignancies [36,37,38,39]. Under inflammatory condition, increased concentration of IL-6 often accompanied an increased concentration of IL-10, a cytokine with anti-inflammatory properties [40]. Previous study in mice also showed that TA promotes TNF-α, IL-1β, and IL-6 reduction [41]. Above all, the results of our study demonstrated that dietary TA from *Galla chinensis* supplementation suppressed systemic and intestinal mucosal inflammatory responses, which might contribute to the improved intestinal development in broilers.

To further explore the mechanisms underlying the inhibition of intestinal inflammatory response by TA from *Galla chinensis* in broiler chickens, TLR4/NF-κB inflammatory pathway genes expressions were examined in this study. In the present study, we found that TA from *Galla chinensis* supplementation inhibited the expression of NF-κB, and no significant changes were observed in the expressions of TLR4 and MyD88 in small intestinal mucosa of broilers. The nuclear factor NF-κB pathway has long been considered a prototypical proinflammatory signaling pathway. The activation of NF-κB can upregulate the expressions of pro-inflammatory cytokines such as TNF-α, IL-1β, and IL-6 [42,43]. Previous study in vitro has demonstrated that TA addition can reverse LPS-induced elevated productions of IL-1β and IL-6 through suppression of NF-κB pathway activation in BV2 microglial cells [44]. Besides, given 20 and 40 mg/kg pure TA by intragastric administration could downregulate IL-6, TNF-α, and NF-κB expressions in the rats challenged by arsenic trioxide [45]. Overall, dietary 300 mg/kg *Galla chinensis* TA supplementation could suppress small intestinal mucosa inflammatory responses potentially through inhibiting NF-κB signaling pathway.

## 5. Conclusions

In conclusion, our study indicated that supplementation of 300 mg/kg microencapsulated TA extracted from *Galla chinensis* showed beneficial effects in improving intestinal development, which might be attributed to the suppression of inflammatory responses in broiler chickens. These findings will aid in our knowledge of the mechanisms through which dietary *Galla chinensis* TA modulates intestinal development and health, as well as support the use of TA sourced from *Galla chinensis* in the poultry industry.

## Figures and Tables

**Table 1:** Ingredients composition and nutrient levels of basal diets (as-fed basis).

Items	Phases	
	0–21 d	21–42 d
Ingredients, %		
Corn	55.91	55.91
Soybean meal, 44% CP	13.78	10.18
Wheat bran	11.98	12.98
Corn starch residue	7.99	9.98
Corn gluten meal	3.99	3.99
Extruded soybean	1.50	2.10
Limestone	1.70	1.70
Calcium monophosphate	1.10	1.10
L-Lysine HCl	1.00	1.00
DL-Methionine	0.20	0.20
L-Threonine	0.10	0.10
Sodium chloride	0.40	0.40
Choline	0.10	0.10
Phytase	0.10	0.10
Complex enzyme	0.02	0.02
Trace mineral premix ^1^	0.10	0.10
Vitamin premix ^2^	0.02	0.02
Antioxidant	0.02	0.02
Total	100	100
Calculated analysis, %		
Metabolizable energy, MJ/kg	12.33	12.50
Crude protein	19.47	17.93
Crude fat	3.45	3.74
Calcium, %	0.94	0.87
Available phosphorus, %	0.35	0.33
Lysine, %	1.15	1.00
Methionine, %	0.50	0.40

^1^ Provided per kilogram of complete basal diet: 10 mg of Cu as CuSO_4_, 100 mg of Fe as FeSO_4_, 1.1 mg of I as Ca(IO_3_)_2_, 65 mg of Zn as ZnSO_4_, 100 mg of Mn as MnSO_4_, and 0.3 mg of Se as Na_2_SeO_3_. ^2^ Provided per kilogram of complete basal diet: vitamin A 10,000 IU, vitamin D_3_ 3000 IU, vitamin E 30 IU, vitamin K_3_ 1.3 mg, vitamin B_1_ 2.2 mg, vitamin B_2_ 8 mg, vitamin B_3_ 8 mg, vitamin B_6_ 4 mg, vitamin B_12_ 0.025 mg, biotin 0.2 mg, niacin 40 mg, folic acid 1 mg, and D-calcium pantothenate 10 mg.

## Data Availability

Not applicable.
